# Replica exchange molecular dynamics simulations reveal the structural and molecular properties of levan-type fructo-oligosaccharides of various chain lengths

**DOI:** 10.1186/s12859-016-1182-7

**Published:** 2016-08-17

**Authors:** Pongsakorn Kanjanatanin, Rath Pichyangkura, Surasak Chunsrivirot

**Affiliations:** 1 Department of Biochemistry, Faculty of Science, Chulalongkorn University, 254 Phaya Thai road, Pathumwan, Bangkok, 10330 Thailand; 2 Structural and Computational Biology Research Group, Department of Biochemistry, Faculty of Science, Chulalongkorn University, 254 Phaya Thai road, Pathumwan, Bangkok, 10330 Thailand

**Keywords:** Levan, Helix, Replica exchange molecular dynamics simulation, Oligosaccharide, Generalized Born implicit solvent

## Abstract

**Background:**

Levan and levan-type fructo-oligosaccharides (LFOs) have various potential applications in pharmaceutical and food industries due to their beneficial properties such as their low intrinsic viscosity and high water solubility. Previous studies showed that they exhibited prebiotic effects, anti-inflammatory and anti-tumor activities against Sarcoma-180 tumor cells of human. Despite their various potential applications, the structural and molecular properties of LFOs of various chain lengths are not well understood.

**Results:**

We employed the replica-exchange molecular dynamics simulations method (REMD) in AMBER14 to elucidate structural and molecular properties of LFOs with chain lengths of 5 (LFO_5_), 10 (LFO_10_) and 15 (LFO_15_) residues in two models of generalized Born implicit solvent (GB_HCT_ and GB_OBC1_). For LFO_10_ and LFO_15_, four distinct conformations (helix-like, partial helix, zig-zag and random structures) were characterized by their upper-middle and lower-middle torsions. For LFO_5_, two distinct conformations (partial helix and random structures) were characterized by their middle torsion and molecular angle of residues 1, 3 and 5. To determine hydrogen bonds important for the formation of helix-like structures of LFO_10_ and LFO_15_, occurrence frequencies of hydrogen bonds were analyzed, and the O6_(i)_--H3O_(i+1)_ hydrogen bond was found with the highest frequency, suggesting its importance in helix formation. Among three dihedral angles between two fructosyl units [ϕ (O5’-C2’-O6-C6), ψ (C2’-O6-C6-C5) and ω (O6-C6-C5-C4)], dihedral angle distributions showed that ω was the most flexible dihedral angle and probably responsible for conformational differences of LFOs.

**Conclusions:**

Our study provides important insights into the structural and molecular properties of LFOs, which tend to form helical structures as the chain length increases from 5 to 15 residues. This information could be beneficial for the selection of LFOs with appropriate lengths and properties for pharmaceutical and biological applications.

**Electronic supplementary material:**

The online version of this article (doi:10.1186/s12859-016-1182-7) contains supplementary material, which is available to authorized users.

## Background

**Fig. 1:**
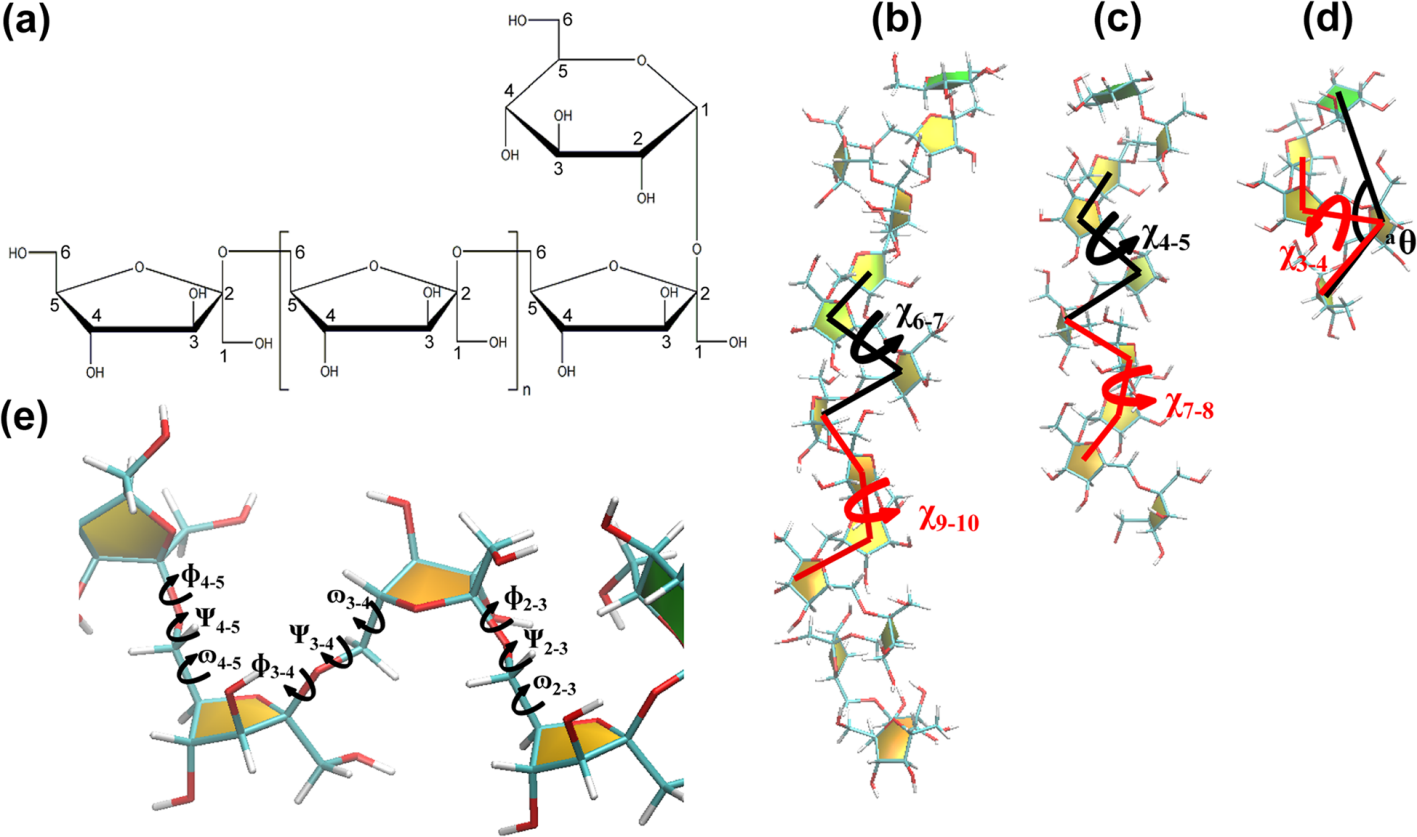
An example of LFO structure and the parameters used in the characterization of the conformations of LFOs. (**a**) An example of LFO structure with no branch. (**b**) The parameters used in the characterization of the conformations of LFOs: the upper-middle (χ_6-7_) and lower-middle (χ_9-10_) torsions for LFO_15_, (**c**) the upper-middle (χ_4-5_) and lower-middle (χ_7-8_) torsions for LFO_10_, (**d**) the molecular angles (θ_a_) and middle torsions (χ_3-4_) for LFO_5_. (**e**) Examples of three dihedral angles between four fructosyl residues of LFO_5_, ω (C4-C5-C6-O6), ψ (C5-C6-O6-C2’) and ɸ (C6-O6-C2’-O5’). The carbon, oxygen and hydrogen atoms were colored in green, red and white, respectively. The six-membered ring of the glucosyl residue and the five-membered rings of fructosyl residue are shown in green and yellow, respectively

Levan-type fructo-oligosaccharides (LFOs) are short chain fructans that contain D-fructofuranosyl residues and are predominantly linked by *β*-(2, 6) linkages in a main chain with some *β*-(2, 1) linked branching points (Fig. 1a). Produced by levansucrase, levan and LFOs are found in various microorganisms such as *Bacillus subtilis* [1], *Zymomonas mobilis* [2], and *Leuconostoc mesenteroides* [3], and play important roles as sources for energy utilization and biofilm formation [4, 5]. The properties of levan and LFOs depend on their chain lengths and branching degrees [6], and they have various desirable properties such as their unusually low intrinsic viscosity [7] and high water solubility [8]. These properties are very beneficial for various industrial applications, especially in food and pharmaceutical industries. For example, levan and LFOs showed prebiotic effects, stimulating the growth of beneficial intestinal bacteria, and also could potentially act as cholesterol lowering agents (MW 2000 kDa) [9, 10]. Furthermore, they could also be served as carbon sources for probiotics such as four strains of *Bifidobacterium sp.* that produce short chain fatty acids, lactate and acetate (MW < 3600 Da) [11]. Moreover, they exhibited anti-inflammatory and anti-tumor activities against Sarcoma-180 tumor cells of human (MW 380–710 kDa) [12, 13]. Despite their various potential applications, the knowledge on the structural and molecular properties of levan and LFOs of various chain lengths is still limited.

Replica exchange molecular dynamics (REMD) method provides an extensive conformational sampling at various temperatures by allowing each replica to exchange their configurations through parallel tempering [14]. Raising the temperature can increase the probability of the system to overcome the energy barrier, consequently enhancing the probability of attaining the global minimum and allowing the sampling of large volumes of phase space. Therefore, the incorporation of higher temperature systems allow the lower temperature systems to access a representative set of the low free energy minima that are accessible by the higher temperature systems [15]. This technique has been used to investigate the properties of oligosaccharides in solution. For example, Re *et al.* employed REMD to elucidate the structural diversity and the changes in conformational equilibria of biantennary complex-type N-glycans [16]. Moreover, Nishima *et al.* used this technique to investigate the effects of bisecting GlcNAc and core fucosylation on conformational properties of biantennary complex-type N-glycans [17]. Recently, Jo *et al.* employed this technique to examine the conformational freedom of the N-glycan core pentasaccharide moiety in solution and found that the conformational variability of the pentasaccharide in solution was more restricted than the *N*-glycan on the protein surface [18]. This method was also employed to investigate conformational flexibility of cellulose oligomers as well as their chain length and temperature dependence [19]. However, to our knowledge, REMD method has not been used in the elucidation of the properties of LFOs of various chain lengths.

In this study, we performed REMD on the models of LFOs with the chain lengths of 5, 10 and 15 residues in two models of generalized Born implicit solvent (GB_HCT_ and GB_OBC1_) to elucidate their structural and molecular properties as well as the relationship between these properties and the chain length. Such information would be beneficial for the selection of LFOs with appropriate lengths and properties for pharmaceutical and biological applications.

## Methods

### Structure preparation and minimization

The structures of LFO_5_, LFO_10_ and LFO_15_ were constructed using the LEaP module in AMBER14 [20], and their atom types and force field parameters were assigned based on GLYCAM06j-1 [21]. Two implicit solvent models (GB_HCT_ and GB_OBC1_) were used in the minimization and simulations of each system. All systems were minimized with 2500 steepest-descent minimization cycles and 2500 conjugate-gradient minimization cycles [22, 23].

### Replica exchange molecular dynamics simulations

Sixteen replicas of each system were initially equilibrated for 500 ps to reach the desired temperature range from 262 to 802 K. REMD of all systems were performed using the SANDER module in AMBER14. Langevin dynamics with a collision frequency of 1 ps^−1^ were used to control the temperatures in all systems. Initial velocity of each system was reseeded by the random number generator [24]. A cut off of 999 Å was used to truncate nonbonded pairs, and the maximum distance of 999 Å between atom pairs was employed to compute the pairwise summation involved in the effective Born radii calculation. All bond-stretching freedoms associated with hydrogen were eliminated by SHAKE algorithm, allowing a time step of 0.002 ps [25]. Each replica was simulated for 100 ns and exchanged every 2 ps. The replicas at 298 K were employed for the analyses of the structural and molecular properties of LFOs with different chain lengths.

To measure the sizes of all systems, their average radii of gyration (ROG) were determined. To determine possible representative structures of LFOs of each chain length, K-means clustering algorithm, as implemented in MMTSB tool sets [26], was employed to cluster the structures from their 100 ns trajectories based on their structural similarities, calculated from their heavy-atom root-mean-square-deviation. To determine a reasonable representative of each cluster, a structure that is most similar to the average structure of all members of each cluster was chosen to be a “centroid;” i.e., a “centroid” is a structure with the lowest heavy-atom root-mean-square-deviation to the average structure. Based on their shapes, these “centroids” were further classified into helix-like, partial helix, zig-zag or random structures as major representative conformers. Helix-like structures were defined as conformations that had more than 1 helical turn, while partial helix structures were defined as conformations that had 1 helical turn. Zig-zag structures were defined as conformations that had zig-zag shapes. Random structures were defined as structures that were not classified as helix-like, partial helix or zig-zag structures.

To plot the free energy maps, various parameters were employed to characterize the structures of LFOs. Since helix-like conformations were observed with high frequencies in LFO_10_ and LFO_15_ and they tended to have similar values of upper-middle and lower-middle torsions, their upper-middle and lower-middle torsions were used to characterize the structures of LFO_10_ and LFO_15_. Their upper-middle torsions were computed by measuring the torsion angles of the centers of masses (CM) of residues 5, 6, 7 and 8 (defined as χ_6-7_ = CM_5_-CM_6_-CM_7_-CM_8_) and residues 3, 4, 5 and 6 (defined as χ_4-5_ = CM_3_-CM_4_-CM_5_-CM_6_) for LFO_15_ and LFO_10_, respectively. Their lower-middle torsions were computed by measuring the torsion angles of CMs of residues 8, 9, 10 and 11 (defined as χ_9-10_ = CM_8_-CM_9_-CM_10_-CM_11_) and residues 6, 7, 8 and 9 (defined as χ_7-8_ = CM_6_-CM_7_-CM_8_-CM_9_) for LFO_15_ and LFO_10_, respectively (Fig. 1b, c). For LFO_5,_ the molecular angles and middle torsions were computed by measuring the angles and torsion angles of CMs of residues 1, 3 and 5 (defined as θ_a_ = CM_1_-CM_3_-CM_5_) and residues 2, 3, 4 and 5 (defined as χ_3-4_ = CM_2_-CM_3_-CM_4_-CM_5_)_,_ respectively (Fig. 1d).

To measure conformational flexibilities, the occurrence frequencies of three dihedral angles between every two fructosyl residues, ω (C4-C5-C6-O6), ψ (C5-C6-O6-C2’) and ϕ (C6-O6-C2’-O5’) (Fig. 1e) were computed. To identify hydrogen bonds important for the formation of helix-like structures of LFO_10_ and LFO_15_, the occurrence frequencies of hydrogen bonds were measured. Only the hydrogen bonds with the occurrence frequency of at least 1 % were used for further analysis.

## Results and discussion

### Reliability of REMD simulations

To determine whether the temperatures were optimally distributed and the number of replicas was sufficient, the acceptance ratios of replica exchange were calculated. The acceptance ratios of the simulations of LFO_15_ in the GB_HCT_ model were almost constant around 28 %, implying a free random walk in the replica (temperature) space (Additional file 1: Figure S1a). Moreover, a free random walk both in the replica space (Additional file 1: Figure S1b) and the temperature space (Additional file 1: Figure S1c) were also confirmed. Furthermore, the canonical probability distribution of the total potential energy at each temperature had sufficient overlap with those of neighbors (Additional file 1: Figure S1d). The results of the REMD simulations of LFO_10_ and LFO_5_ in the GB_HCT_ model were also similar, and their average acceptance ratios were almost constant around 37 and 50 % for LFO_10_ and LFO_5_, respectively. For the systems simulated in the GB_OBC1_ model, the results of REMD simulations were also similar to those simulated in the GB_HCT_ model, and their average acceptance ratios were almost constant around 28, 36 and 50 % for LFO_15_, LFO_10_ and LFO_5_, respectively. These results indicate good reliability of the REMD simulations of all systems.

### Sizes of LFOs

**Fig. 2:**
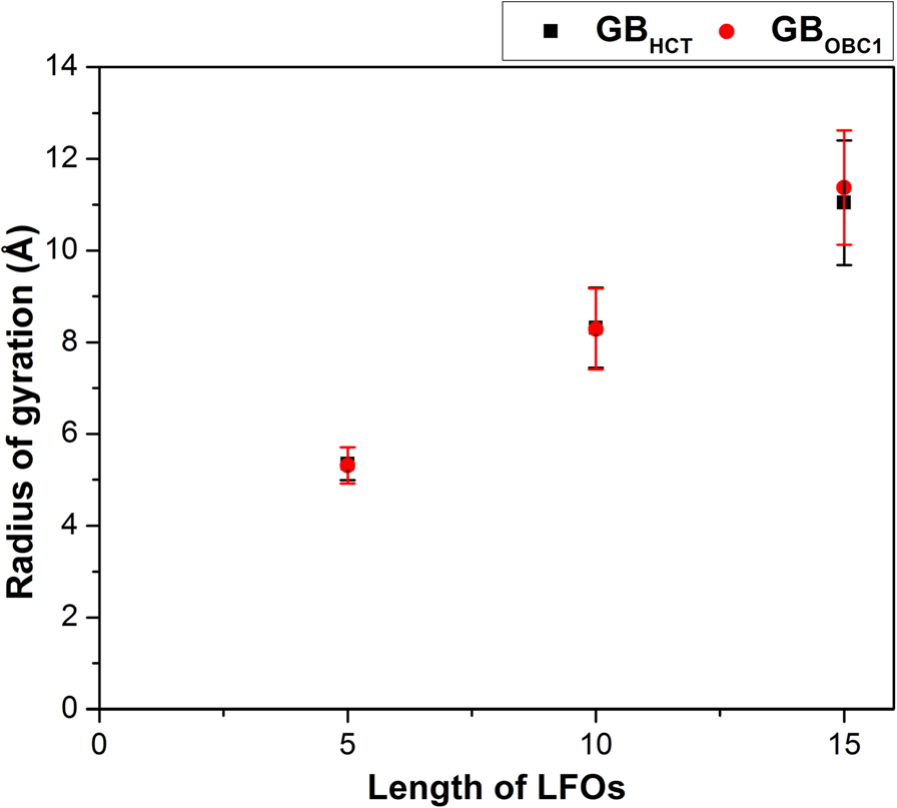
The average radii of gyration calculated from the heavy atoms of LFOs simulated in GB_HCT_ (square) and GB_OBC1_ (dot) models

The sizes of LFOs were determined by measuring their radii of gyration. Figure 2 shows that the trends of the radii of gyration of LFOs simulated in the GB_HCT_ model and those simulated in the GB_OBC1_ model are similar. The radii of gyration of LFOs tended to increase as their chain lengths increased from 5 to 15 residues. These results suggest the extension of the structures of LFOs as their chain lengths increase.

### Conformations of LFO_15_, LFO_10_ and LFO_5_

**Fig. 3:**
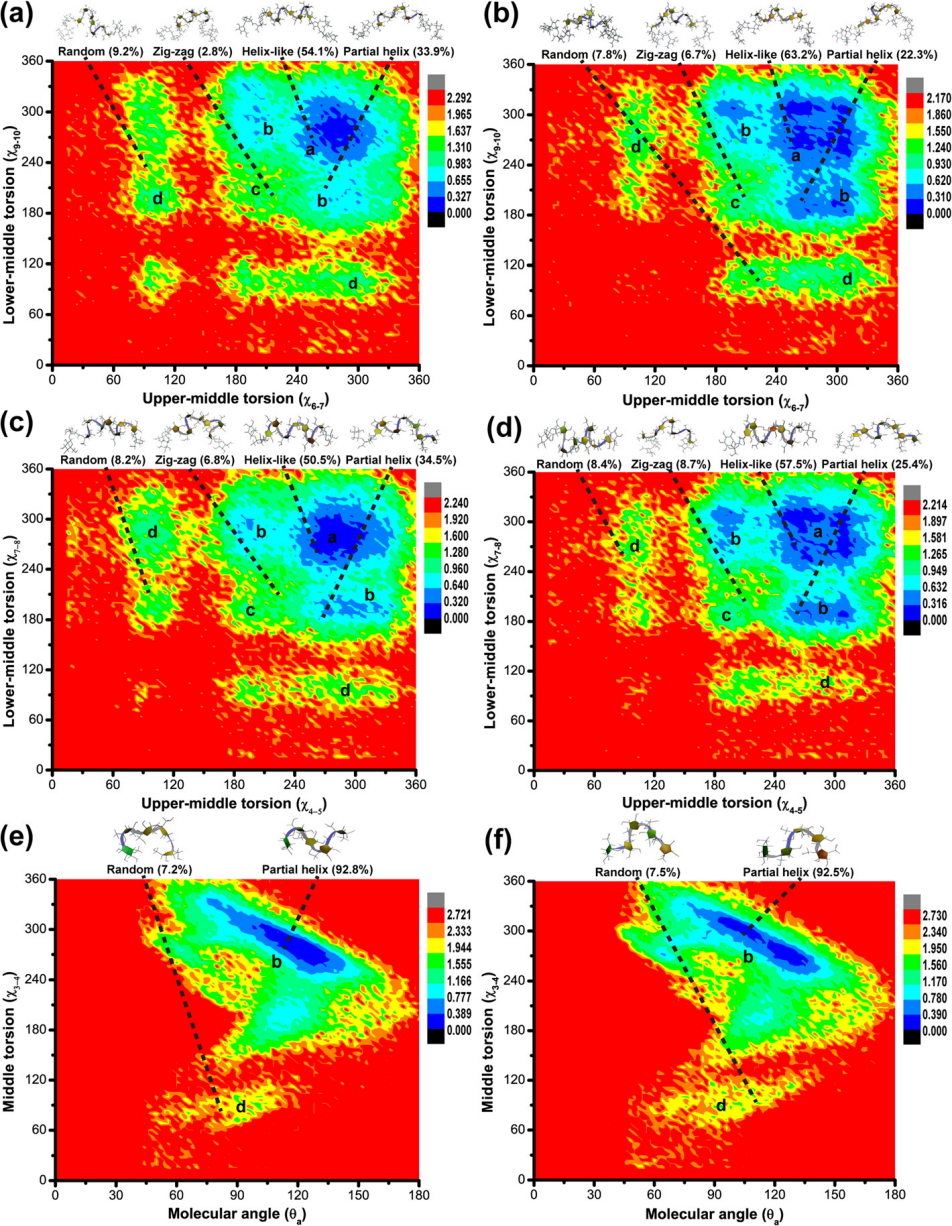
The relative free energy (kcal/mol) maps of LFO_15_ (**a**), LFO_10_ (**c**) and LFO_5_ (**e**) simulated in the GB_HCT_ model as well as those of LFO_15_ (**b**), LFO_10_ (**d**) and LFO_5_ (**f**) simulated in the GB_OBC1_ model. The groups a, b, c and d are helix-like, partial helix, zig-zag and random structures. Their major representative conformers and populations are also shown

Figure 3 shows the free-energy maps of LFO_15_, LFO_10_ and LFO_5_ as simulated in GB_HTC_ and GB_OBC1_ models as well as their major representative conformers and their population sizes from clustering analysis and centroid classification. For LFO_15_, four major conformations such as helix-like (a), partial helix (b), zig-zag (c) and random (d) structures were observed after clustering analysis and centroid classification (Fig. 3a, b and Additional file 1: Figure S2 and Figure S5), and they were characterized by their upper-middle and lower-middle torsions (χ_6-7_ and χ_9-10_). Helix-like structures were found with the highest population of 54.1 and 63.2 % for those simulated in GB_HCT_ and GB_OBC1_ models, respectively. Helix-like structures took up conformations of left-handed 3-fold helices and tended to have their upper-middle and lower-middle torsions in the similar range of around 240–315°. The conformations with the second highest population were partial helix structures, and their population sizes were 33.9 and 22.3 % for systems simulated in GB_HCT_ and GB_OBC1_ models, respectively. The other two conformations were zig-zag and random structures. Zig-zag structures were found with the population sizes of 2.8 and 6.7 % for systems simulated in GB_HCT_ and GB_OBC1_ models, respectively. The population sizes of random structures simulated in GB_HCT_ and GB_OBC1_ models were 9.2 and 7.8 %, respectively.

Similar to the conformations of LFO_15_, four major conformations such as helix-like (a), partial helix (b), zig-zag (c) and random (d) structures were found for LFO_10_ after clustering analysis and centroid classification (Fig. 3c, d and Additional file 1: Figure S3 and Figure S6). These conformations were characterized by their upper-middle and lower-middle torsions (χ_4-5_ and χ_7-8_). The conformation with the highest population sizes of 50.5 and 57.5 % was helix-like structures for those simulated in GB_HCT_ and GB_OBC1_ models, respectively. Partial helix structures occurred with the second highest population sizes of 34.5 and 25.4 % for those simulated in GB_HCT_ and GB_OBC1_ models, respectively. The population sizes of zig-zag structures were 6.8 and 8.7 % and those of random structures were 8.2 and 8.4 % for systems simulated in GB_HCT_ and GB_OBC1_ models, respectively.

For LFO_5_, two major conformations such as partial helix (b) and random (d) structures were observed after clustering analysis and centroid classification, probably due to its shorter chain length as compared to those of LFO_10_ and LFO_15_ (Fig. 3e, f and Additional file 1: Figure S4 and Figure S7). These conformations were characterized by their molecular angles (θ_a_) and middle torsion (χ_3-4_). Partial helix structures were observed with the population sizes of 92.8 and 92.5 % for those simulated in GB_HCT_ and GB_OBC1_ models, respectively. Random structures were also found with the population sizes of 7.2 and 7.5 % for those simulated in GB_HCT_ and GB_OBC1_ models, respectively.

Table 1 shows the populations of major representative conformers of LFO_15_, LFO_10_ and LFO_5_ simulated in GB_HCT_ and GB_OBC1_ models as determined from clustering analysis and centroid classification. As the chain length increased, the population of the helix-like structures tended to increase. These results may suggest that LFOs have tendencies to form helices as their chain lengths are extended.

**Table 1:** The populations of major representative conformers of LFO_15_, LFO_10_ and LFO_5_ simulated in GB_HCT_ and GB_OBC1_ models as determined from clustering analysis and centroid classification

Solvent model	Major representative conformer	Population (%)		
		LFO_15_	LFO_10_	LFO_5_
GB_HCT_	Helix-like structure	51.4	50.5	-
	Partial helix structure	33.9	34.5	92.8
	Zig-zag structure	2.8	6.8	-
	Random structure	9.2	8.2	7.2
GB_OBC1_	Helix-like structure	63.2	57.5	-
	Partial helix structure	22.3	25.4	92.5
	Zig-zag structure	6.7	8.7	-
	Random structure	7.8	8.4	7.5

### Hydrogen bonds important for the formation of helix-like structures

**Fig. 4:**
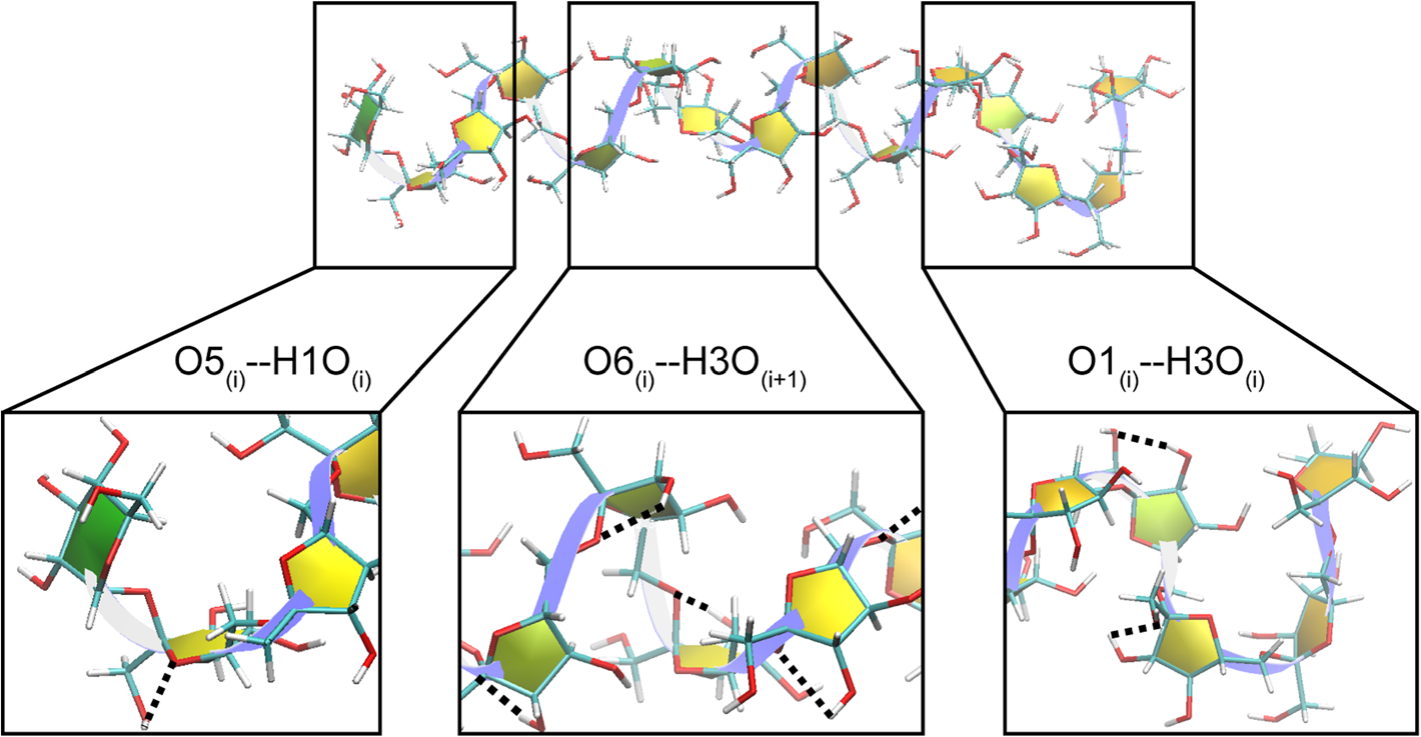
Hydrogen bonds important for the formation of helix-like structures (LFO_15_ simulated in the GB_HCT_ model is shown as an example). Middle; the O6_(i)_--H3O_(i+1)_ hydrogen bond (occurrence frequency = 65.0 %). Right; the O1_(i)_--H3O_(i)_ hydrogen bond. (occurrence frequency = 15.4 %). Left; the O5_(i)_--H1O_(i)_ hydrogen bond (occurrence frequency = 11.5 %). Hydrogen bonds are represented as dash lines. The LFO chain and fructosyl units are represented as ribbon and filled yellow color representations, respectively

To elucidate the hydrogen bonds important for the formation of helix-like structures, the occurrence frequencies of hydrogen bonds in helix-like structures of LFO_15_ and LFO_10_ with the occurrence frequencies of at least 1 % were analyzed. For the systems simulated in the GB_HCT_ model, the O6_(i)_--H3O_(i+1)_ hydrogen bonds (between residue i and i + 1) were found with the highest frequency, and their glycosidic oxygens acted as important hydrogen bond acceptors that interacted with the hydroxyl groups of C3 atoms of the furanose rings and probably helped stabilize the helix-like structures (Table 2 and Fig. 4). The hydrogen bonds with the second and third highest occurrence frequencies for both LFO_15_ and LFO_10_ were the O1_(i)_--H3O_(i)_ and O5_(i)_--H1O_(i)_ hydrogen bonds, which were the hydrogen bonds within the same residue (Table 2 and Fig. 4). The trends of the occurrence frequencies of the hydrogen bonds of LFO_15_ and LFO_10_ in the GB_OBC1_ model were also similar to those in the GB_HCT_ model (Table 2). These three hydrogen bonds (O6_(i)_--H3O_(i+1),_ O1_(i)_--H3O_(i)_ and O5_(i)_--H1O_(i)_ hydrogen bonds), especially the O6_(i)_--H3O_(i+1)_ hydrogen bond that was found with the highest frequency, are probably important for the formation of helix-like structures of LFO_15_ and LFO_10_ as their occurrence frequencies are higher than other hydrogen bonds.

**Table 2:** Occurrence frequencies of hydrogen bonds found in helix-liked structures of LFO_15_ and LFO_10_

Solvent model	Residue that form a hydrogen bond	Type	Occurrence frequency^a^ (%)	
			LFO_15_	LFO_10_
GB_HCT_	i, i	O1_(i)_--H3O_(i)_	15.4	15.3
		O5_(i)_--H1O_(i)_	11.5	12.5
	i, (i + 1)	O6_(i)_--H3O_(i+1)_	65.0	60.2
GB_OBC1_	i, i	O1_(i)_--H3O_(i)_	10.0	10.0
		O5_(i)_--H1O_(i)_	8.3	7.8
	i, (i + 1)	O6_(i)_--H3O_(i+1)_	37.5	34.8

^a^Only hydrogen bonds with the occurrence frequency of at least 7 % are shown

### Conformational flexibilities

**Fig. 5:**
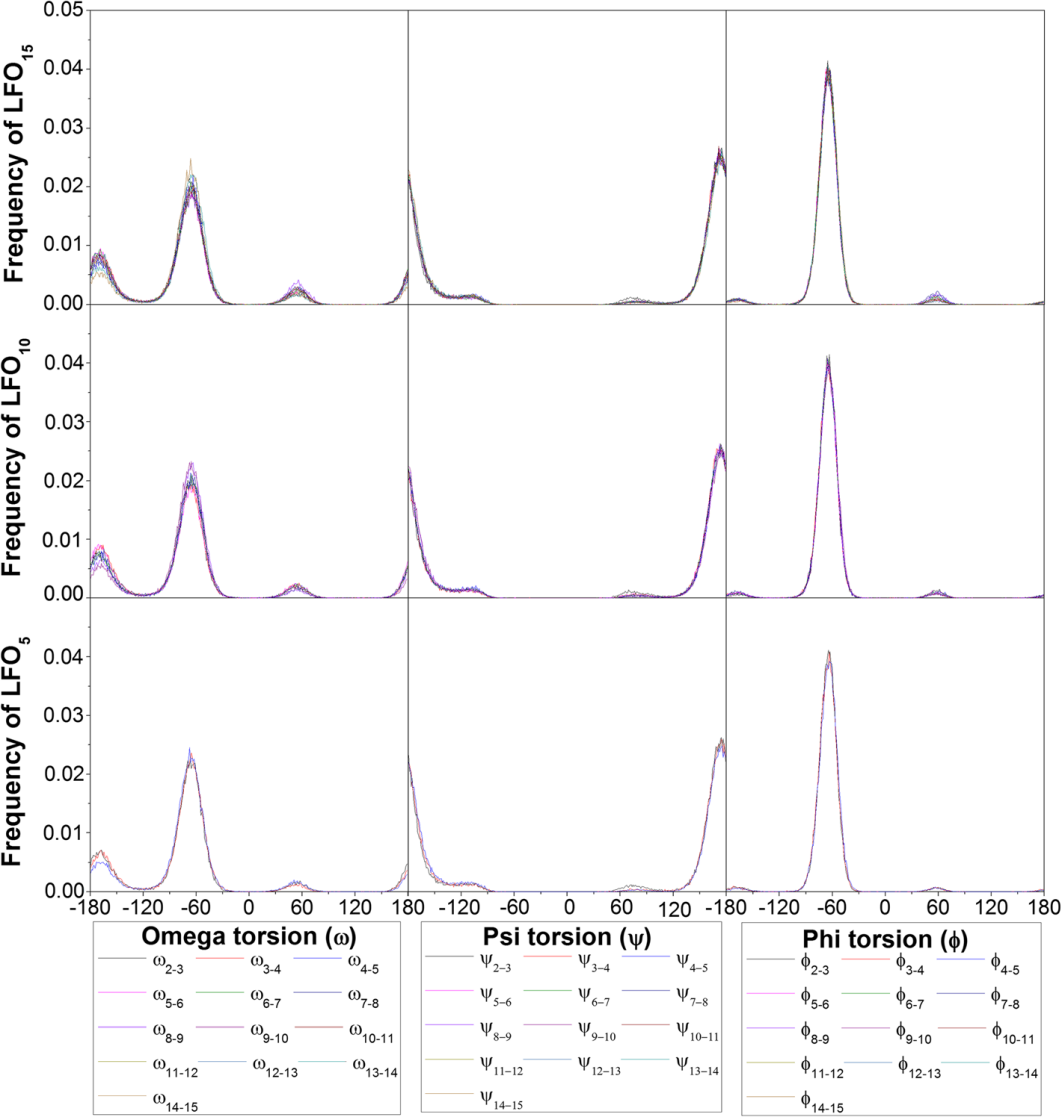
The frequencies of the three dihedral angles of all glycosidic linkage of LFO_15_, LFO_10_ and LFO_5_ in the GB_HCT_ model. Each dihedral angle is shown in different color

To investigate the conformational flexibilities of LFO_15,_ LFO_10_ and LFO_5_, the occurrence frequencies of ω, ψ and ɸ of all glycosidic bonds were measured. For the systems simulated in the GB_HCT_ model, ψ and ɸ of all glycosidc linkages of all LFOs exhibited single major peaks around 173° and -63°, respectively (Fig. 5). However, ω was more flexible than ψ and ɸ as it exhibited one major peak and two minor peaks (Fig. 5). The results from the systems simulated in the GB_OBC1_ model were similar (Additional file 1: Figure S8); ω exhibited more peaks and was more flexible than ψ and ɸ. These results suggest that the flexibility of ω may be responsible for the conformational diversity of LFOs since this dihedral angle has more possibilities in rotating and changing the conformations of LFOs.

## Conclusions

To elucidate the structural and molecular properties of LFOs as well as the relationship between these properties and their chain lengths, REMD were performed on systems of LFO_5_, LFO_10_ and LFO_15_ in GB_HCT_ and GB_OBC1_ solvent models. We found that as the chain length increased, the radii of gyration tended to increase, suggesting the extension of the conformations as the chain length increases. After clustering analysis and centroid classifications, four major representative conformations (helix-like, partial helix, zig-zag and random structures) were found for LFO_15_ and LFO_10,_ while two conformations (partial helix and random structures) were identified for LFO_5_. The free energy maps show that the four conformations of LFO_15_ and LFO_10_ were characterized by their upper-middle and lower-middle torsions, whereas the two conformations of LFO_5_ were characterized by their molecular angles and middle torsions. As the chain length increased from 5 to 15 residues, the conformation populations of the helix-like structures tended to increase, suggesting the possible tendency of LFOs to form helices as their chain lengths are extended. Moreover, the O6_(i)_--H3O_(i+1)_ hydrogen bond was found with the highest frequency, suggesting its importance in helix formation of LFO_15_ and LFO_10_. Furthermore, ω was found to be more flexible than ψ and ɸ and probably responsible for the conformational diversity of LFOs. This study gives important insights into the structural and molecular properties of LFOs; they tend to form helical structures as the chain length increases from 5 to 15 residues. Our findings may be useful in the selection of LFOs with appropriate chain lengths and structural properties for pharmaceutical and biological applications.

## Additional file

Additional file 1: Figure S1. (a) The acceptance ratio of replica exchange of the adjacent pairs of the simulations of LFO_15_ in the GB_HCT_ model. (b) Replica exchange at 298 K. (c) Time series of temperature exchange of three arbitrary chosen replicas 2 (*black*), 8 (*red*) and 16 (*blue*). (d) The canonical probability of the total potential energy of the systems at 16 temperatures simulated in the GB_HCT_ model. **Figure S2.** The “centroid” structure of each cluster of LFO_15_ simulated in the GB_HCT_ model. Their conformation types and populations are also shown. **Figure S3.** The “centroid” structure of each cluster of LFO_10_ simulated in the GB_HCT_ model. Their conformation types and populations are also shown. **Figure S4.** The “centroid” structure of each cluster of LFO_5_ simulated in the GB_HCT_ model. Their conformation types and populations are also shown. **Figure S5.** The “centroid” structure of each cluster of LFO_15_ simulated in the GB_OBC1_ model. Their conformation types and populations are also shown. **Figure S6.** The “centroid” structure of each cluster of LFO_10_ simulated in the GB_OBC1_ model. Their conformation types and populations are also shown. **Figure S7.** The “centroid” structure of each cluster of LFO_5_ simulated in the GB_OBC1_ model. Their conformation types and populations are also shown. **Figure S8.** The frequencies of the three dihedral angles of all glycosidic linkage of LFO_15_, LFO_10_ and LFO_5_ in the GB_OBC1_ model. Each dihedral angle is shown in different color. (DOCX 4740 kb)

## Abbreviations

GB: Generalized Born implicit solvent
LFOs: Levan-type fructo-oligosaccharides
REMD: Replica-exchange molecular dynamics simulations.

## References

[1] Steinmetz M, Le Coq D, Aymerich S (1985). The DNA sequence of the gene for the secreted *Bacillus subtilis* enzyme levansucrase and its genetic control sites. Mol Gen Genet.

[2] Goldman D, Lavid N, Schwartz A (2008). Two Active Forms of *Zymomonas mobilis* Levansucrase: An ordered microfibril structure of the enzyme promotes levan polymerization. J Biol Chem.

[3] Kang HK, Seo MY, Seo ES (2005). Cloning and expression of levansucrase from *Leuconostoc mesenteroides* B-512 FMC in *Escherichia coli*. Biochim Biophys Acta.

[4] Dawes E, Ribbons D (1966). Sucrose utilization by *Zymomonas mobilis*: formation of a levan. Biochem J.

[5] Dogsa I, Brloznik M, Stopar D (2013). Exopolymer diversity and the role of levan in *Bacillus subtilis* biofilms. PLoS ONE.

[6] Srikanth R, Reddy CHS, Siddartha G (2015). Review on production, characterization and applications of microbial levan. Carbohydr Polym.

[7] Arvidson SA, Rinehart BT, Gadala-Maria F (2006). Concentration regimes of solutions of levan polysaccharide from *Bacillus sp*. Carbohydr Polym.

[8] Han YW (1990). Microbial levan. Adv Appl Microbiol.

[9] Gibson GR, Probert HM, Van Loo J (2004). Dietary modulation of the human colonic microbiota: updating the concept of prebiotics. Nutr Res Rev.

[10] Yamamoto Y, Takahashi Y, Kawano M (1999). In vitro digestibility and fermentability of levan and its hypocholesterolemic effects in rats. J Nutr Biochem.

[11] Marx SP, Winkler S, Hartmeier W (2000). Metabolization of β-(2, 6)-linked fructose-oligosaccharides by different bifidobacteria. FEMS Microbiol Lett.

[12] Rairakhwada D, Pal A, Bhathena Z (2007). Dietary microbial levan enhances cellular non-specific immunity and survival of common carp (Cyprinus carpio) juveniles. Fish Shellfish Immunol.

[13] Yoo S-H, Yoon EJ, Cha J (2004). Antitumor activity of levan polysaccharides from selected microorganisms. Int J Biol Macromolec.

[14] Sugita Y, Okamoto Y (1999). Replica-exchange molecular dynamics method for protein folding. Chem Phys Lett.

[15] Earl DJ, Deem MW (2005). Parallel tempering: Theory, applications, and new perspectives. Phys Chem Chem Phys.

[16] Re S, Miyashita N, Yamaguchi Y (2011). Structural diversity and changes in conformational equilibria of biantennary complex-type N-glycans in water revealed by replica-exchange molecular dynamics simulation. Biophys J.

[17] Nishima W, Miyashita N, Yamaguchi Y (2012). Effect of bisecting GlcNAc and core fucosylation on conformational properties of biantennary complex-type N-glycans in solution. J Phys Chem B.

[18] Jo S, Qi Y, Im W (2016). Preferred conformations of N-glycan core pentasaccharide in solution and in glycoproteins. Glycobiology.

[19] Shen T, Langan P, French AD (2009). Conformational flexibility of soluble cellulose oligomers: chain length and temperature dependence. J Am Chem Soc.

[20] Case D, Babin V, Berryman J, et al. AMBER 14, 2014. University of California, San Francisco

[21] Kirschner KN, Yongye AB, Tschampel SM (2008). GLYCAM06: a generalizable biomolecular force field. Carbohydrates J Comput Chem.

[22] Hawkins GD, Cramer CJ, Truhlar DG (1996). Parametrized models of aqueous free energies of solvation based on pairwise descreening of solute atomic charges from a dielectric medium. J Phys Chem.

[23] Onufriev A, Bashford D, Case DA (2000). Modification of the generalized Born model suitable for macromolecules. J Phys Chem B.

[24] Cerutti DS, Duke R, Freddolino PL (2008). A vulnerability in popular molecular dynamics packages concerning Langevin and Andersen dynamics. J Chem Theory Comput.

[25] Ryckaert J-P, Ciccotti G, Berendsen HJ (1977). Numerical integration of the cartesian equations of motion of a system with constraints: molecular dynamics of n-alkanes. J Comput Phys.

[26] Feig M, Karanicolas J, Brooks CL (2004). MMTSB Tool Set: enhanced sampling and multiscale modeling methods for applications in structural biology. J Mol Graph Model.

