# Supplementary material for: Replica exchange molecular dynamics simulations reveal the structural and molecular properties of levan-type fructo-oligosaccharides of various chain lengths
Source: BMC Bioinformatics. 2016 Aug 17;17:306. doi: 10.1186/s12859-016-1182-7 (PMC4989353; doi:10.1186/s12859-016-1182-7)
Supplement: Additional file 1: Figure S1. — (a) The acceptance ratio of replica exchange of the adjacent pairs of the simulations of LFO15 in the GBHCT model. (b) Replica exchange at 298 K. (c) Time series of temperature exchange of three arbitrary chosen replicas 2 (black), 8 (red) and 16 (blue). (d) The canonical probability of the total potential energy of the systems at 16 temperatures simulated in the GBHCT model. Figure S2. The “centroid” structure of each cluster of LFO15 simulated in the GBHCT model. Their conformation types and populations are also shown. Figure S3. The “centroid” structure of each cluster of LFO10 simulated in the GBHCT model. Their conformation types and populations are also shown. Figure S4. The “centroid” structure of each cluster of LFO5 simulated in the GBHCT model. Their conformation types and populations are also shown. Figure S5. The “centroid” structure of each cluster of LFO15 simulated in the GBOBC1 model. Their conformation types and populations are also shown. Figure S6. The “centroid” structure of each cluster of LFO10 simulated in the GBOBC1 model. Their conformation types and populations are also shown. Figure S7. The “centroid” structure of each cluster of LFO5 simulated in the GBOBC1 model. Their conformation types and populations are also shown. Figure S8. The frequencies of the three dihedral angles of all glycosidic linkage of LFO15, LFO10 and LFO5 in the GBOBC1 model. Each dihedral angle is shown in different color. (DOCX 4740 kb) [file 12859_2016_1182_MOESM1_ESM.docx]

Replica exchange molecular dynamics simulations reveal the structural and molecular properties of levan-type fructo-oligosaccharides of various chain lengths

Pongsakorn Kanjanatanin,^1,2^ Rath Pichayangkura,^1^ and Surasak Chunsrivirot^1,2,*^

^1^Department of Biochemistry, Faculty of Science, Chulalongkorn University, Bangkok, 10330, Thailand

^2^Structural and Computational Biology Research Group, Department of Biochemistry, Faculty of Science, Chulalongkorn University, Bangkok, 10330, Thailand

Pongsakorn Kanjanatanin; email: pongsakorn.ka@student.chula.ac.th

Rath Pichayangkura; email: prath@chula.ac.th

Surasak Chunsrivirot; email: surasak.ch@chula.ac.th

*Corresponding author:

Department of Biochemistry, Faculty of Science, Chulalongkorn University, 254 Phayathai road, Pathumwan, Bangkok 10330

Email: surasak.ch@chula.ac.th

Additional files 1: Fig. S1-S8

**
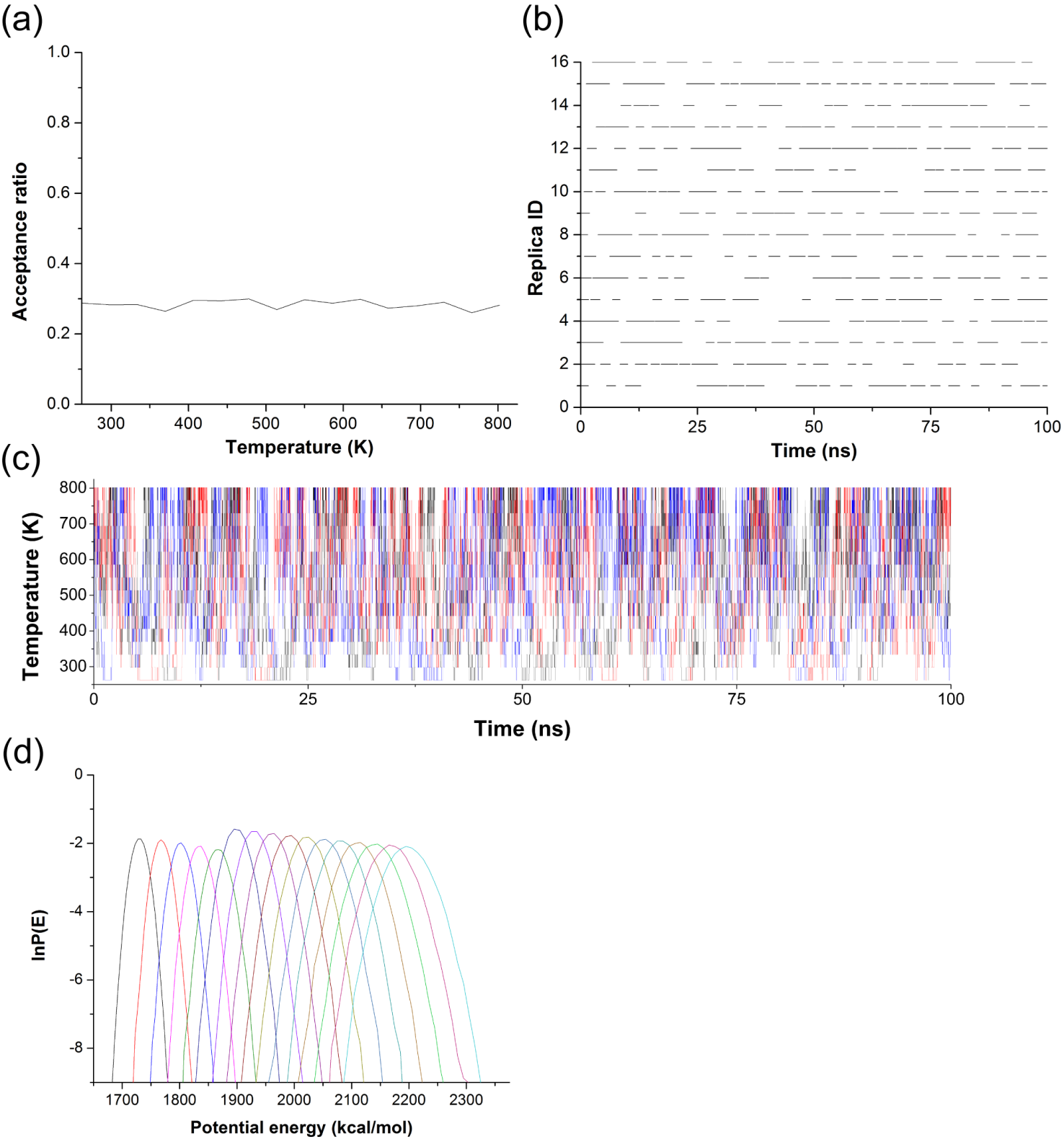
**

**Fig S1. (a)** The acceptance ratio of replica exchange of the adjacent pairs of the simulations of LFO_15_ in the GB_HCT_ model. **(b)** Replica exchange at 298 K. **(c)** Time series of temperature exchange of three arbitrary chosen replicas 2 (black), 8 (red) and 16 (blue). **(d)** The canonical probability of the total potential energy of the systems at 16 temperatures simulated in the GB_HCT_ model.

**
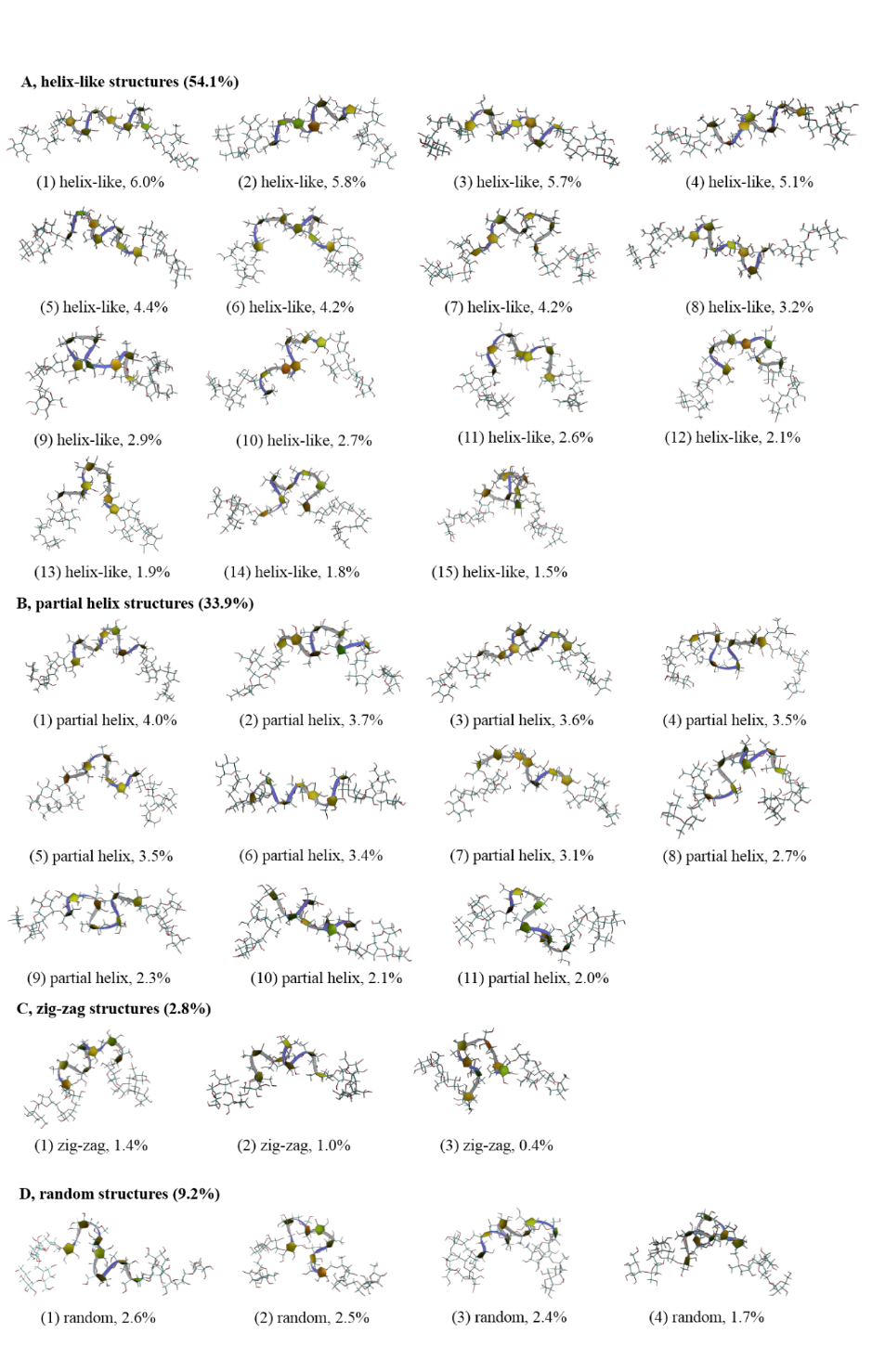
**

**Fig S2.** The “centroid” structure of each cluster of LFO_15_ simulated in the GB_HCT_ model. Their conformation types and populations are also shown.

**
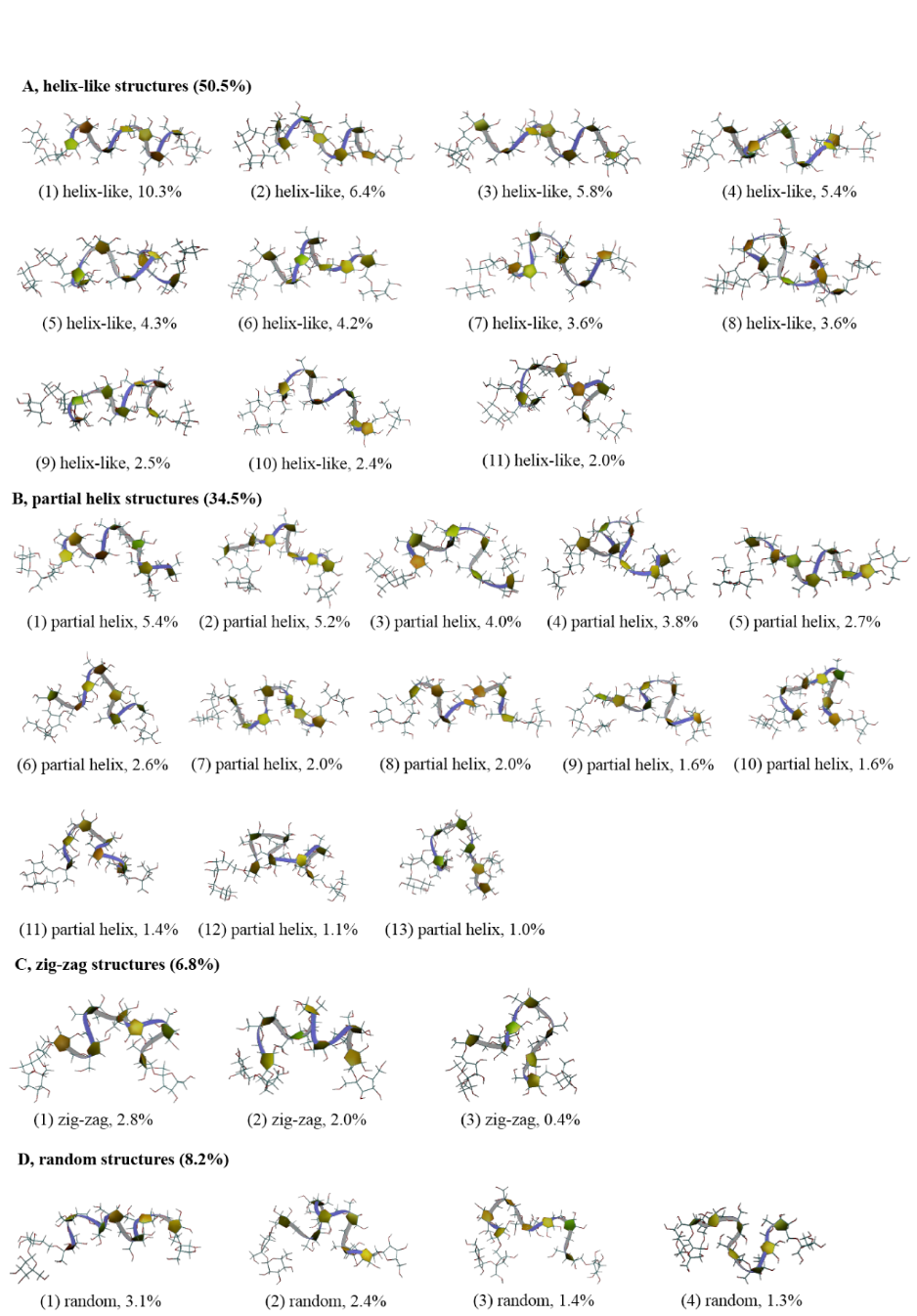
**

**Fig S3.** The “centroid” structure of each cluster of LFO_10_ simulated in the GB_HCT_ model. Their conformation types and populations are also shown.

**
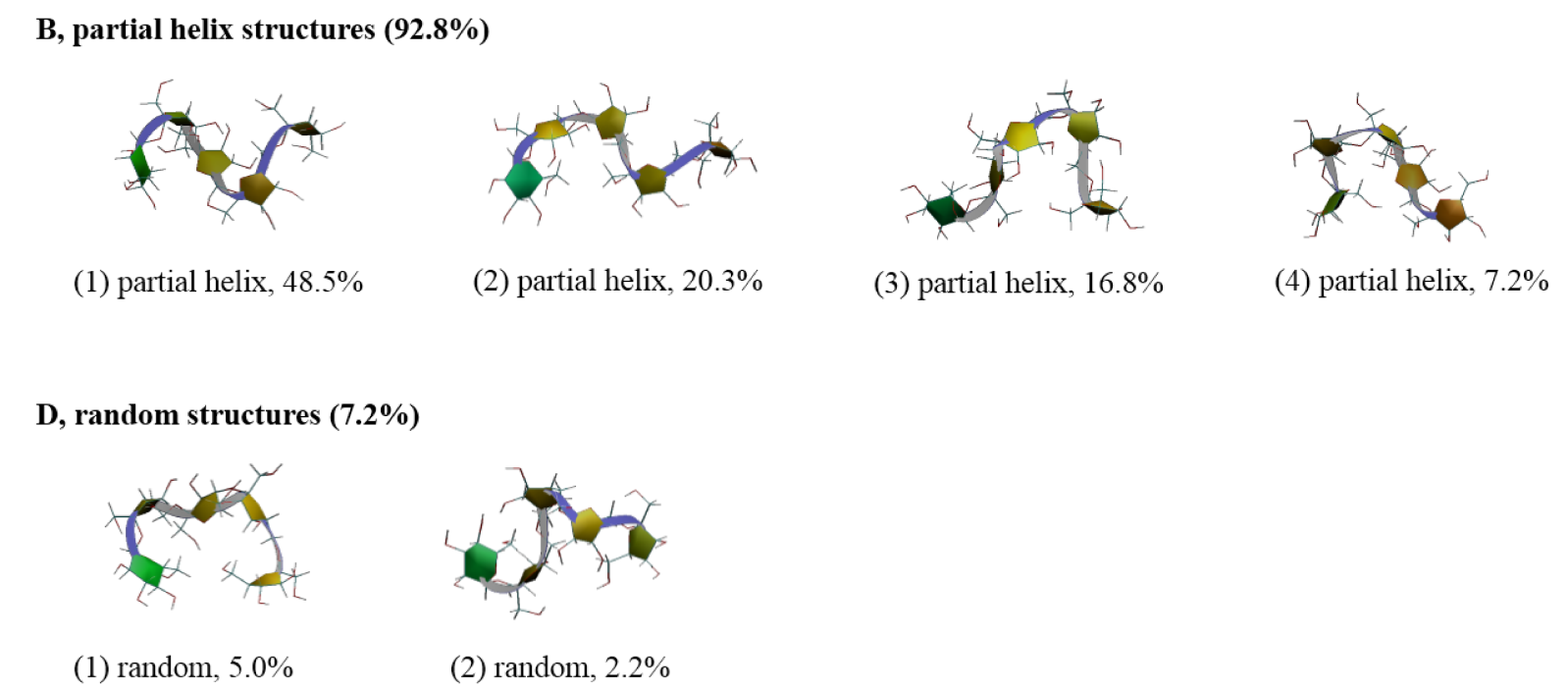
**

**Fig S4.** The “centroid” structure of each cluster of LFO_5_ simulated in the GB_HCT_ model. Their conformation types and populations are also shown.

**
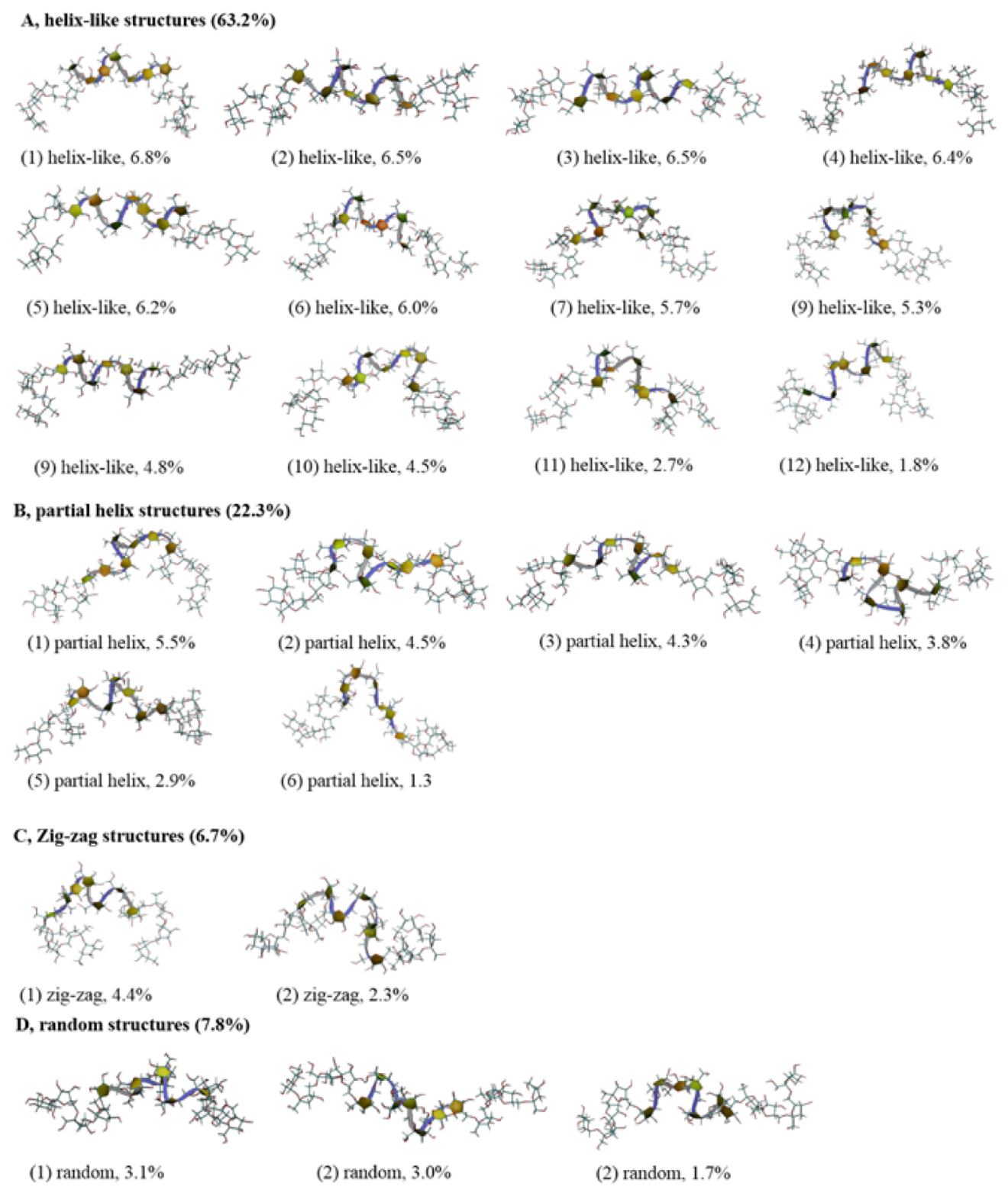
**

**Fig S5.** The “centroid” structure of each cluster of LFO_15_ simulated in the GB_OBC1_ model. Their conformation types and populations are also shown.

**
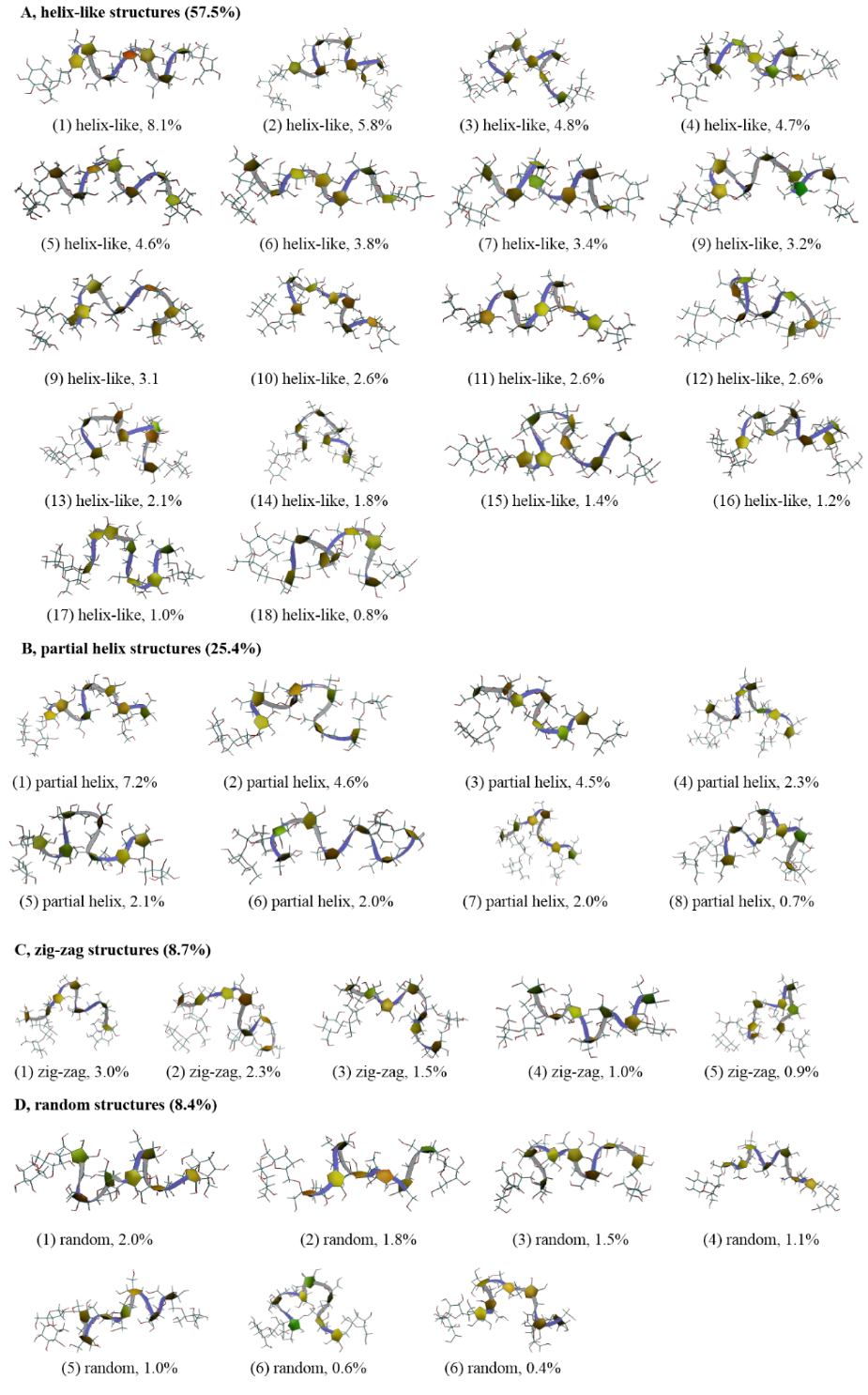
**

**Fig S6.** The “centroid” structure of each cluster of LFO_10_ simulated in the GB_OBC1_ model. Their conformation types and populations are also shown.

**
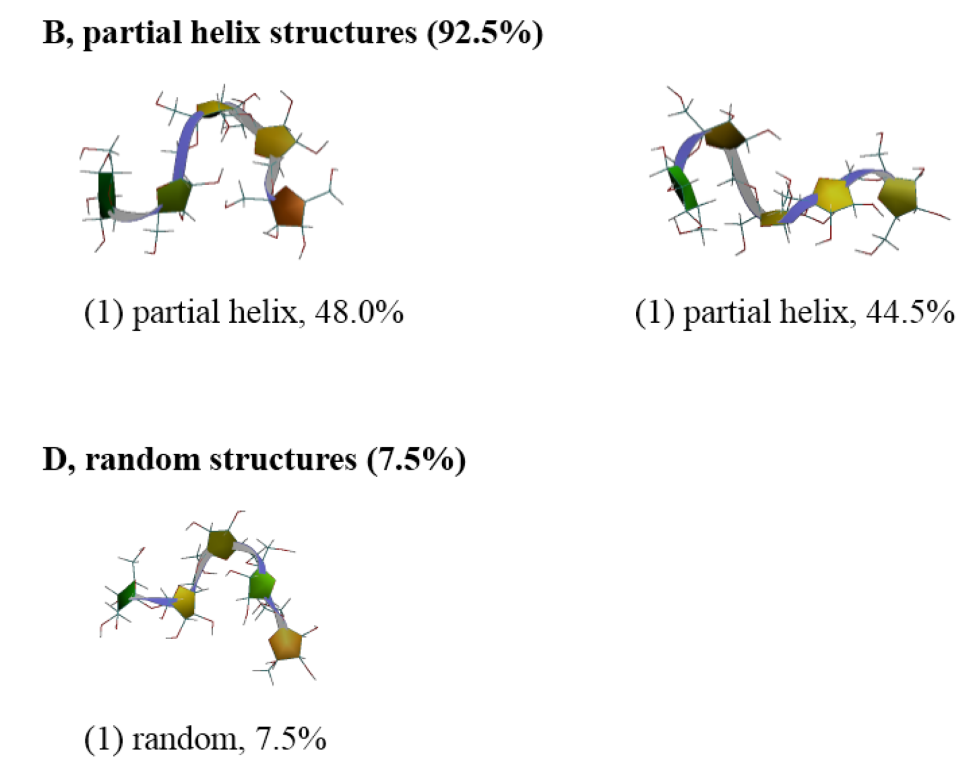
**

**Fig S7.** The “centroid” structure of each cluster of LFO_5_ simulated in the GB_OBC1_ model. Their conformation types and populations are also shown.

**
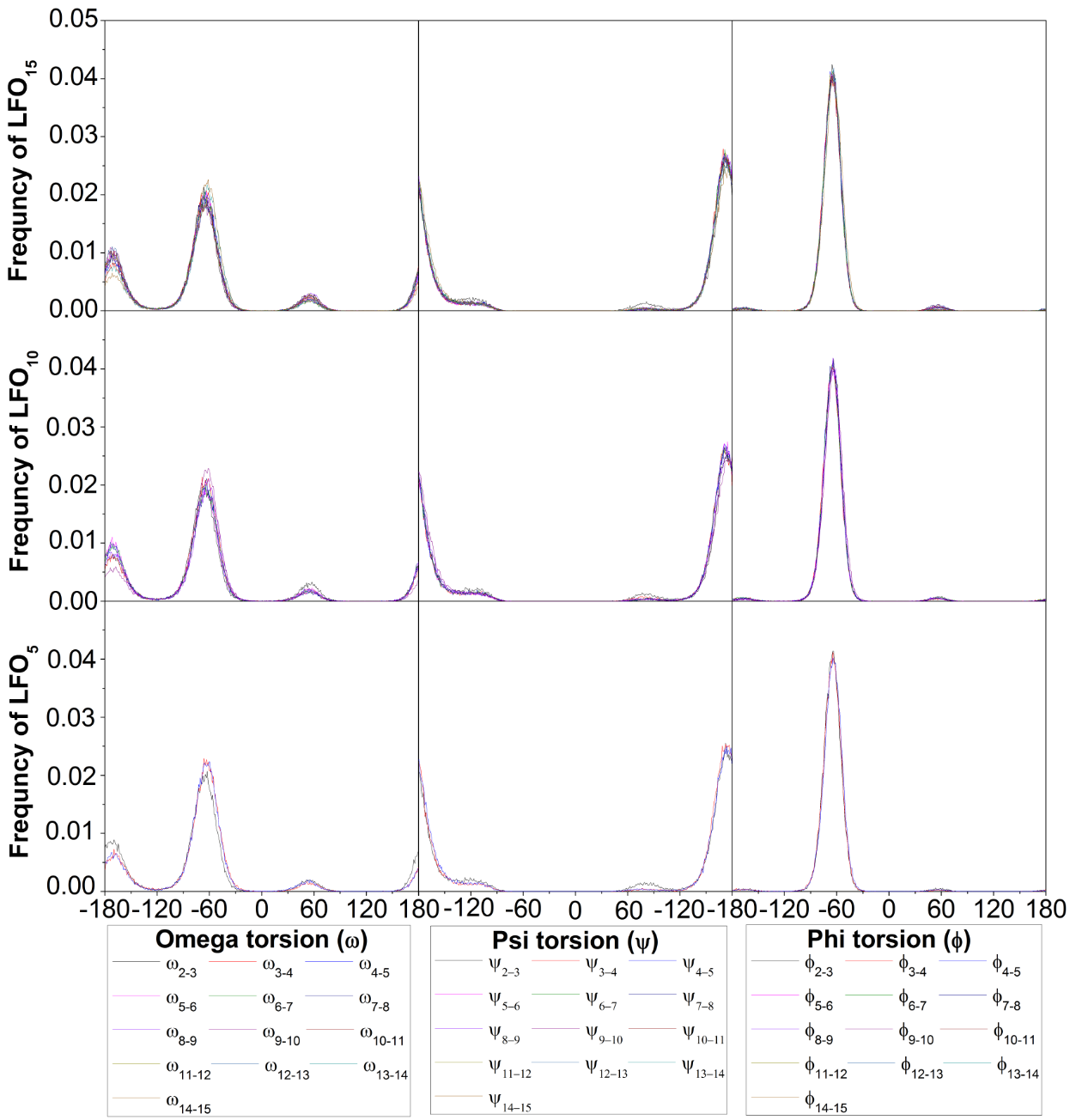
**

**Fig S8.** The frequencies of the three dihedral angles of all glycosidic linkage of LFO_15_, LFO_10_ and LFO_5_ in the GB_OBC1_ model. Each dihedral angle is shown in different color.
